# Exploring Lignin Conformation in Organic and Deep Eutectic Solvents Using Small-Angle Neutron Scattering

**DOI:** 10.1021/acs.langmuir.5c03558

**Published:** 2026-01-02

**Authors:** Subramee Sarkar, Maggie Kroon, Daniel Papp, Nicolas Martin, Charlotta Turner, Karen J. Edler

**Affiliations:** † Centre for Analysis and Synthesis, Department of Chemistry, 5193 Lund University, Naturvetarvägen 24, Lund 223 62, Sweden; ‡ Laboratoire Léon Brillouin, CEA, CNRS, Université Paris-Saclay, CEA Saclay, 91191 Gif-sur-Yvette, France; # Wallenberg Initiative Materials Science for Sustainability, Centre for Analysis and Synthesis, Department of Chemistry, Lund University, Naturvetarvägen 24, Lund 223 62, Sweden

## Abstract

Lignin, a structurally intricate and heterogeneous phenolic biopolymer, holds considerable promise as a sustainable alternative to petrochemical-derived materials across diverse applications in the energy and materials sectors. However, precise lignin molecular weight and structure determination remains challenging due to its intrinsic tendency to aggregate in solution and the absence of chemically analogous polymer standards for chromatographic techniques. By employing small-angle neutron scattering, this study aims at precise measurement of lignin’s polymeric conformation, aggregation behavior, and radius of gyration in organic gel permeation chromatography/NMR solvent, tetrahydrofuran (THF), and in an emerging class of solvent systems known as deep eutectic solvents (DES). These “designer” solvents, formed from tailored hydrogen bond donors and acceptors, are gaining importance for lignin extraction from biomass and analytical characterization. However, their influence on lignin conformation in solutions remains unexplored. Our study reveals that both organosolv and Indulin AT kraft lignin in THF exhibit loosely associated polymeric conformations. Upon D_2_O addition, Indulin AT undergoes moderate swelling, suggestive of partial dissolution, while organosolv lignin undergoes substantial elongation with directional ordering, resulting in flexible rod-like structures. Lignin oil from a reductive catalytic fractionation process (RCF), in contrast, remains well-dispersed in THF and shows minimal structural change with solvent polarity modulation via D_2_O addition. Indulin AT and organosolv lignin solvated in the choline chloride/oxalic acid/ethylene glycol DES adopt dense, cylindrical morphologies. These structures show moderate temperature sensitivity and notable resistance to D_2_O-induced structural perturbation, highlighting strong lignin–DES interactions. Additionally, lignin extracted from cocoa bean shells using a diol-based DES and subsequently dissolved in the same solvent demonstrates a fractal-like morphology, which evolves with D_2_O content and temperature, revealing a complex solvation landscape. These results offer molecular-level insight into lignin’s solvent-dependent structural transitions, enabling more accurate molecular weight estimation and supporting optimization of lignin processing for high-performance biobased formulations and advanced materials.

## Graphical abstract

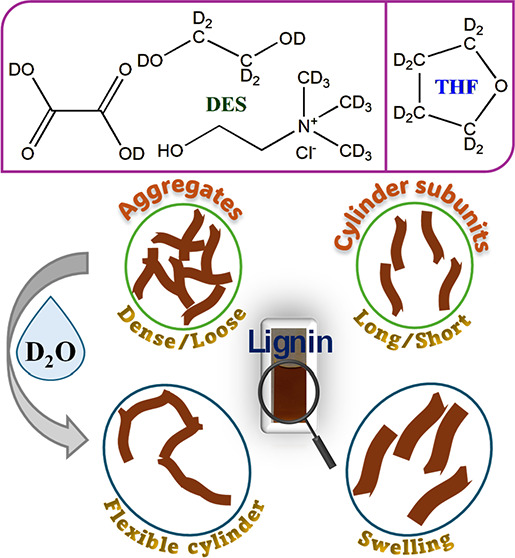

## 1 Introduction

Lignin, the only naturally occurring aromatic biopolymer, is a complex, amorphous, and heterogeneous macromolecule predominantly located in the secondary cell walls of vascular plants. Lignin acts as a structural glue binding cellulose and hemicellulose in lignocellulosic biomass and plays a crucial role in imparting mechanical strength, hydrophobicity, and resistance to microbial attack. It is composed of three primary phenylpropane units (syringyl, guaiacyl, and *p*-hydroxyphenyl), which are cross-linked by ether and carbon–carbon bonds (β-O-4′, β-5′, 5–5′, 4-O-5′, β-1′, and β–β′).^1,2^ The presence of aromatic rings and diverse functional groups in lignin has driven considerable research interest toward its conversion into high-value chemicals, polymer additives, biofuels, and functional materials, providing a promising sustainable alternative to petroleum-based chemicals.^3^ Globally, an estimated 50–70 million tons of technical lignins (kraft lignin, lignosulfonate, organosolv, and soda lignin) are produced annually as a byproduct of pulping and biorefinery processes, offering significant potential for valorization rather than being incinerated solely for its heat value.^4^ In addition to conventional lignocellulosic feedstocks, lignin can also be sourced from agricultural and food-processing residues, offering low-cost sustainable routes to support the circular bioeconomy.

Despite its potential, lignin’s structural variability, introduced through both the biomass source and extraction methods, leads to differences in molecular weight, polydispersity, and functional group composition, posing significant challenges to broader application. Its inherent heterogeneity, strong intermolecular interactions, and limited solubility in common solvents complicate efforts to characterize and functionalize it. Thus, a fundamental understanding of lignin’s molecular conformation and aggregation behavior in solution is critical for enabling efficient utilization. To investigate these properties, computational methods as well as a range of spectroscopic (ultraviolet, Fourier-transform infrared, Raman, nuclear magnetic resonance (NMR) spectroscopy), scattering (dynamic light scattering (DLS), multiangle laser light scattering (MALLS), small-angle X-ray scattering (SAXS), small-angle neutron scattering (SANS)), and chromatographic methods (size exclusion chromatography (SEC), gel permeation chromatography (GPC), gel-filtration chromatography (GFC)) are being employed.^5–10^

A key focus in most literature studies lies in determining lignin’s average molecular weight and molecular weight distribution, typically via SEC. However, due to the lack of chemically analogous lignin calibration standards, SEC often yields inaccurate values, with potential errors spanning several hundred Daltons. This issue is further complicated by lignin’s propensity to self-aggregate in solution. Recent work by Papp et al. compared molecular weight values obtained from GPC and pulsed field gradient diffusion NMR (pfg-diffusion NMR) for kraft, organosolv, and reductive catalytic fractionation (RCF) oil lignin, using tetrahydrofuran (THF) as the solvent in both methods. In addition, pfg-diffusion NMR was performed in alternative solvents such as dimethylformamide (DMF) and dimethyl sulfoxide (DMSO); however, these results are not directly comparable to GPC data due to solvent-dependent differences in lignin aggregation behavior.^11^ These findings underscore the need for alternative approaches to accurately determine lignin’s solution-state conformation and aggregation behavior.

In this context, SAXS and SANS are powerful, nondestructive techniques for probing the nanoscale structure of macromolecules in solution. SAXS provides valuable information about the molecular assembly of polymers based on electron density differences; however, its contrast can be limited for certain lignin polymer–solvent combinations. On the other hand, using deuterated solvents enhances the scattering contrast between solvent and hydrogenated lignin species in SANS, which enables detailed investigation of lignin’s size, shape, and degree of aggregation in various environments. Previous studies have utilized SANS, often in conjunction with NMR, to explore the solution behavior of lignin derived from diverse sources. For example, Zhao et al. (2017) investigated alkaline lignin in DMSO-*d*_6_ and NaOD, highlighting that lignin with fewer aliphatic hydroxyl groups solubilized more readily based on functional group–solution structure correlations.^12^ Yang et al. (2019) studied kraft lignin in deuterated ethylene glycol (EG-d6), revealing insights into solvent-dependent aggregation where EG-d6 facilitated lignin association.^13^ Zhang et al. (2022) examined lignin from switchgrass, miscanthus, sorghum, corncob, eucalyptus, and pine in DMSO-*d*_6_, NaOD, and EG-d6, finding that lignin aggregates are likely composed of higher-molecular-weight lignin with a greater aliphatic-to-phenolic hydroxyl ratio. Also, a discrepancy in the radius between SANS and DLS was observed for lignin nanoparticles in aqueous solutions, with DLS measurements showing hydrodynamic radii about three times larger than those from SANS, indicating the presence of a substantial solvation shell around the nanoparticles.^14^ Ratnaweera et al. (2015) compared lignin from softwood, hardwood, and wheat straw, each characterized by distinct cross-linking densities, dissolved in DMSO-*d*_6_, and found that methoxy content and source-specific functional groups significantly influenced the formation of well-defined cylindrical aggregates in that solvent.^15^ In a related study, Imel et al. (2016) examined the effect of poly­(ethylene oxide) (PEO) additives in DMSO-*d*_6_ on the assembly of lignin from the same biomass sources, demonstrating that PEO promotes anisotropic aggregation, primarily through the elongation of cylindrical domains.^16^ Zhang et al. (2024) applied both in situ and ex situ SANS to monitor structural transformations of organosolv poplar lignin in deuterated methanol (MeOH-*d*_4_) during solvolysis, both in the absence and presence of a copper-based catalyst. They noticed that lignin transitions from a rigid cylindrical conformation at room temperature to a more spherical, flexible, and folded structure at 250 °C, with a copper-containing porous metal oxide catalyst enhancing the formation and condensation of smaller lignin particles (50 Å).^17^

Advancing this line of research, the present study aims to elucidate the solution-state conformation, molecular dimensions, and solvation effects in both a conventional GPC solvent, THF, and in novel deep eutectic solvents (DES), which are rapidly gaining attention for lignin extractions. DES are mixtures of two or more species where strong intermolecular interactions depress the melting point below that of the ideal mixture, allowing formation of room-temperature liquids even if the components are solids.^18^ Recent findings suggest that diol-based DES can preserve the β-O-4 linkages in lignin, retaining more native-like, larger polymeric structures, while higher organic acid contents in the DES cause greater breakdown of the polymer.^19^ Despite the growing interest in DES, the molecular-level understanding of lignin–DES interactions and their influence on lignin’s structural organization in solution remains unexplored. In this study, SANS has been employed to investigate the structure of three lignin systems, as previously studied by Papp et al., i.e., Indulin AT, organosolv, and RCF oil, to probe these lignins’ true solution behavior and provide calibration support for NMR and GPC-based measurements. Also, DES-extracted lignin derived from cocoa bean shells (CBS), a byproduct of the chocolate industry, has been included to compare its solution behavior and morphology with those of other lignin types.

The primary objectives of this work are to (i) compare lignin’s conformation in a conventional organic GPC solvent vs DES-based solvent, (ii) evaluate the effect of D_2_O-induced polarity changes on lignin–solvent interactions, (iii) assess temperature-dependent structural transitions in DES medium, and (iv) correlate the dimensions of cylindrical lignin obtained from SANS with molecular weights derived from pfg-diffusion NMR measurements. Solvent composition, polarity, and temperature are hypothesized to influence lignin conformation, aggregation, and hierarchical organization, which can be captured using SANS combined with appropriate model fitting. A better understanding of such processing parameters in appropriate solvents can guide solvent choice for processing of lignin materials, and comparison between different techniques can highlight where each characterization method is useful, or any potential pitfalls in measuring complex systems such as lignin solutions.

## 2 Experimental Methods

### 2.1 Materials

The lignin samples used in this study included organosolv lignin (from Ola Wallberg, Lund University, Sweden), Indulin AT kraft lignin (from Ingevity, South Carolina, USA), and birch sawdust-derived lignin oil from reductive catalytic fractionation (from Joseph Samec, Stockholm University, Sweden). All these lignin samples were used as received, without further purification. Information regarding the characterization of these lignins using pfg-diffusion NMR and GPC in various solvents is available in the prior literature.^11^ The other type of lignin sample used was extracted from CBS, sourced from Mondelez International (UK) using two DES systems, choline chloride/oxalic acid/ethylene glycol and xylitol/citric acid/ethylene glycol. The resulting lignin samples are referred to in this work as CBS extract-COE and CBS extract-XCE, respectively. The choline chloride/oxalic acid/ethylene glycol DES was prepared by mixing choline chloride (≥98%), oxalic acid (98%, anhydrous), and ethylene glycol (99%), purchased from Thermo Fischer Scientific, in a 1:0.2:2 molar ratio. The mixture was heated to 50 °C under stirring until a homogeneous, clear liquid was obtained. This DES, at the chosen molar ratio, was selected for both lignin extraction and dissolution in the SANS experiments as it is reported to effectively inhibit undesired lignin recondensation during extraction to yield lignin with a high β-O-4 content and better structural preservation.^19^ For comparison, CBS was also extracted and investigated in a more sustainable diol-based DES, consisting of xylitol (99%), citric acid (≥99%), and ethylene glycol (99%) (Thermo Fischer Scientific). These were combined in a 1:1:2 molar ratio and heated to 80 °C until a homogeneous liquid formed. The CBS was milled into a fine powder to obtain a mean particle diameter of 473 μm using a 24-series Circ-U-Flow hammer mill (Schutte, USA). The isolation of CBS extract-COE was carried out in a Monowave 200 microwave (Anton Paar, UK) by heating 5% solid-to-liquid to 80 °C for 30 min, whereas CBS extract-XCE was obtained using conventional heating on a hot plate at 120 °C for 6 h using the same solid-to-liquid ratio (5%). Further details of the extraction protocol are described elsewhere.^20^ Deuterated solvents employed for SANS studies included D_2_O (Sigma-Aldrich, St. Louis, MO, USA, 99.9 atom % D), deuterated tetrahydrofuran (THF-d8) (Sigma-Aldrich, ≥99% purity, ≥99.5 atom % D), and a deuterated DES system comprising choline chloride-d9 (Sigma-Aldrich, ≥98% purity, 98 atom % D), oxalic acid-d2 (Aldrich Chemical Co., Inc., Milwaukee, Wisconsin, USA, 99 atom % D), and ethylene glycol-d6 (Thermo Fischer Scientific, Waltham, MA, USA, 98% purity, 98.9 atom % D). The deuterated DES, 1:0.2:2 d9-choline chloride/d2-oxalic acid/d6-ethylene glycol, referred to as d-COE, was prepared from the components as described earlier for the hydrogenated species. For the deuterated xylitol/citric acid/ethylene glycol DES, d-XCE, deuterated xylitol and citric acid were prepared via exchanging hydrogens with excess D_2_O. Using this method, 90.4% of the exchangeable hydrogens in xylitol and 97.8% in citric acid were replaced by deuterium, based on NMR measurements. These were then combined with d6-ethylene glycol in a 1:1:2 molar ratio to prepare the DES. Dynamic Light Scattering (DLS) measurements were conducted using a Nano-ZS Zetasizer instrument (Malvern Instruments Ltd., UK). All measurements were performed in triplicate at room temperature with a detection backscatter angle of 173°. pfg-diffusion NMR experiments were carried out at 25 °C on a Bruker Avance III HD 500 MHz spectrometer (Bruker, Billerica, MA, USA), with the detailed experimental setup and data processing provided elsewhere.^11^

### 2.2 SANS Measurements and Data Analysis

SANS experiments were performed on the SAM instrument, operated by the Laboratoire Léon Brillouin at the Institut Laue-Langevin (ILL).^21^ The samples were measured using a neutron wavelength of 6 Å, covering a Q-range of 0.005 to 0.4 Å^–1^, which allowed for probing lignin structures across different length scales. Each sample was loaded into 1 mm path-length quartz cells, and scattering data were collected for 30 min per sample, ensuring sufficient data resolution for structural analysis. Background subtraction was performed using corresponding solvent blanks. Four distinct lignin systems, organosolv, Indulin AT, RCF oil, and CBS-extracted lignin, were dissolved at 5, 10, and 20 mg/mL in THF-d8 and/or deuterated DES, depending on solubility, with D_2_O contents varied at 0, 5, and 10% w/w. Specifically, RCF oil was dissolved only in THF-d8, and CBS-extracted lignin was dissolved only in the deuterated DES. Measurements for the THF-based systems were conducted at a constant temperature of 25 °C. For the DES-based systems, a temperature-dependent study was performed at intervals between 25 and 55 °C. Small-angle X-ray scattering data were also recorded for the organosolv, Indulin AT, and RCF oil lignin samples in the COE DES (Figure S1, Table S1), showing good reproducibility of scattering data from these systems. Although scattering was performed at three different concentrations, the results from the 20 mg/mL samples has been mostly used for comparison as it provided a better signal-to-noise ratio, particularly in THF-based systems. Scattering in 5 mg/mL samples was usually too weak for reliable analysis. The neutron scattering length density (SLD) for the different components, used for the fitting is provided in Table S2.

The following model functions were employed for the analysis of the SANS data:

1 For the majority of the samples, the scattering data were fitted in SASView 5.06 (http://www.sasview.org/) using an empirical model that combines contributions from individual lignin subunits and larger aggregates, represented, respectively, by a cylindrical form factor and a power law term, as used previously for lignin solutions in the work of Yang et al.^13^ and Zhang et al.^14^ The intensity of the scattering is fitted as

1 $$I\left(q\right)=\frac{A}{q^{n}}+BP\left(q\right)+bkg$$

where the term *A*/*q* ^*n*^ represents the power law arising from scattering from aggregates, with the power exponent (*n*) reflecting the compactness of the structures; *P*(*q*) denotes the form factor of a rigid cylinder to describe lignin subunits; the constants A and B are proportional to the number densities of aggregates and subunits, respectively; and bkg accounts for incoherent background scattering. The size of the lignin aggregates is often large (microns), so these are mostly outside of the measurable range of the SANS instrument. Only the tail of that scattering is observed and is approximated by the *A*/*q* ^*n*^ term. Power law scattering at low Q in SANS can be attributed to a range of structures reflecting scattering from swollen polymer chains for values near 5/3 to Gaussian chains for values of 2 and to clustered networks, where the compactness of the structures gives a power law corresponding to the mass fractal dimension with values between 2 and 3. For larger structures, surface fractal scattering for rough surfaces gives exponents from 3 (rough) up to 4 (smooth).^22^ Values above 4 are strictly unphysical but are frequently observed for polydisperse hierarchically structured systems with high local concentrations, resulting in contributions to the power law from scattering at many length scales.^23^

The cylindrical form factor, characterized by a cross-sectional radius R and length L, is expressed as

2 $$P\left(q\right)=\int_{0}^{\pi /2}{\left|F\left(q,\theta \right)\right|}^{2}\sin\theta \;d\theta$$

where $F\left(q,\theta \right)=2\left(\rho_{cyl}-\rho_{solv}\right)V_{cyl}j_{0}\left(qH\cos\theta \right)\left[\frac{J_{1}\left(qR\sin\theta \right)}{qR\sin\theta }\right]$ , where *H* = *L*/2 is half the cylinder length, *j*_0_(*x*) = sin *x*/*x*, *J*_1_ is the first-order cylindrical Bessel function, *V*_cyl_ = π*R* ^2^ *L*, and θ is the angle between the cylinder axis and the scattering vector *q*.

Assuming a Schulz distribution of cross-section radius *R*

3 $$f\left(R\right)=\frac{R^{z}}{\Gamma \left(z+1\right)}{\left(\frac{z+1}{\left\langle R\right\rangle }\right)}^{z+1}\exp\left[\frac{-R\left(z+1\right)}{\left\langle R\right\rangle }\right]$$

4 $$The\;polydispersity\;\sigma \;is\;given\;by{\;\sigma }^{2}=1/\left(z+1\right)$$

The averaged form factor is

5 $$\overset{®}{P\left(q\right)}=\int P\left(q\right)f\left(R\right)\;dr$$

An illustration of the separate contributions of the power law and cylinder form factor fits for organosolv lignin in deuterated THF and d9-choline chloride/d2-oxalic acid/d6-ethylene glycol is provided in Figure S2.

2 In the case of CBS-extracted lignin, the scattering profiles were fitted using the fractal cylinder model implemented via the NCNR SANS Analysis package in Igor Pro.^24^ The scattering function of this model describes aggregates of rigid cylinders organized with a mass fractal structure, used by Zhao et al.^12^ for modeling SANS data from technical lignins in solution and is given by^12,25^

6 I(q)=nP(Q){1+[S(q)−1]}+bkg

7 $$S\left(q\right)=1+\frac{1}{{\left(qR\right)}^{D}}\frac{D\Gamma \left(D-1\right)}{{\left[1+1/q^{2}\xi^{2}\right]}^{\left(D-1\right)/2}}\times \sin\left[\left(D-1\right)\tan\left(q\xi \right)\right]$$

where *D* denotes the fractal dimension reflecting the self-similar structure of the aggregates; *R* is the radius of the cylindrical subunit; ξ is the correlation length or upper cutoff of the fractal regime; and Γ is the gamma function. *P*(*q*) again refers to the form factor of a rigid cylinder and n is the number density of such subunits. In this model, variations in *D* and ξ provide insight into how lignin molecules organize in solution, where a higher *D* with a larger ξ indicates more compact but extended aggregate networks, while lower values reflect more dispersed or loosely associated smaller structures.

3 For organosolv lignin in THF in the presence of D_2_O, the scattering data could not be captured by either rigid cylinder model so were fitted using the flexible cylinder model available in SASView 5.06 (http://www.sasview.org/). The form factor for this model was developed through empirical fits to Monte Carlo simulations of semiflexible linear chains representative of wormlike micelles. It is parametrized by the contour length of the cylinder (*L*_c_) and its persistence length (*l*_p_), the latter often represented as the Kuhn length 2 × l_p_, capturing the chain stiffness.^26,27^

## 3 Results and Discussion

### 3.1 Influence of Solvent on Lignin Conformation

The SANS scattering curves for Indulin AT, organosolv lignin, RCF oil, and CBS-extracted lignin in both THF and DES (Figure 1) revealed notable differences in their structural organization and aggregation behavior. In most cases, we could observe an upturn in the low-q region of the SANS profiles, indicating the presence of larger, supramolecular lignin aggregates, which only account for a fraction of the total structure. Scattering in the intermediate- to high-q regions is dominated by cylindrical structures that have been attributed to lignin subunits. In many earlier studies, lignin subunits have been defined as the smallest individual entities within the lignin structure, typically corresponding to the lowest-molecular-weight fractions identified by GPC or the smallest-size components detected by light scattering, SAXS, or SANS. These subunits are often modeled as short cylindrical particles, with radii and lengths falling within this size range.^14–16,28,29^ The scattering intensity for organosolv lignin exhibited higher scattering than Indulin AT at the same concentration, suggesting a greater degree of aggregation or structural compactness in organosolv lignin under identical solvent conditions. Notably, Indulin AT and organosolv lignin both form elongated cylindrical subunits in DES, while in THF, these lignins form shorter cylinders (Table 1). The power law exponent is close to that for aggregates assembled in a polymer-coil-like morphology for organosolv lignin in THF, while for Indulin AT, the value of 0.7 ± 0.1 is close to the value of 1.0 expected for isolated cylinders. Meanwhile, RCF oil displayed the lowest scattering intensity at low Q, cylinder dimension and power exponent value in THF, pointing to a highly dispersed (well-dissolved) system with minimal self-association. The slightly higher scattered intensity at high q is attributed to local density fluctuations within the smaller subunits, rather than aggregation, and is consistent with the RCF oil being more fully solvated in THF. As the size of a single monolignol unit reported in the literature is ∼8 Å,^13^ the cylinder radius observed for RCF oil closely matches that of an individual monolignol. In contrast, the larger radii observed for Indulin AT, organosolv, and CBS-extracted lignin suggest that their cylindrical subunits are composed of at least two monolignol units stacked or associated laterally. Thus, the structural building block on SANS length scales is the same in all the three lignins (Indulin AT, organosolv and RCF oil), represented by a cylindrical morphology, with the main differences arising from the size distribution of the lignin subunits and the association or self-assembly behavior, which markedly depends on the lignin chemical structure, composition, and functional groups (aliphatic hydroxyl, phenolic hydroxyl, methoxyl, and carboxyl content) as well as solvent–lignin interactions. The higher power exponent values (2.8–4.4) in DES indicate that lignin molecules assemble into denser, more packed cylindrical structures in this solvent, whereas THF promotes extended, loosely associated, and more solvated structures. The exponent slightly above 4 at low q in the case of organosolv lignin is above the theoretical value of 4.0, which defines sharp interfaces, reflecting effective slopes in the experimental data influenced by the complex hierarchical aggregate structure in these solutions.^23^ To test the robustness of the analysis, the SANS data of organosolv lignin in d-COE were also fitted using the same model, with the power-law exponent constrained to the Porod limit of 4 (Figure S3). The resulting fit shows slight deviations in the crossover between large-scale aggregates and subunit scattering, as expected for this hierarchical system, but these do not significantly alter the extracted structural parameters.

**Figure 1:**
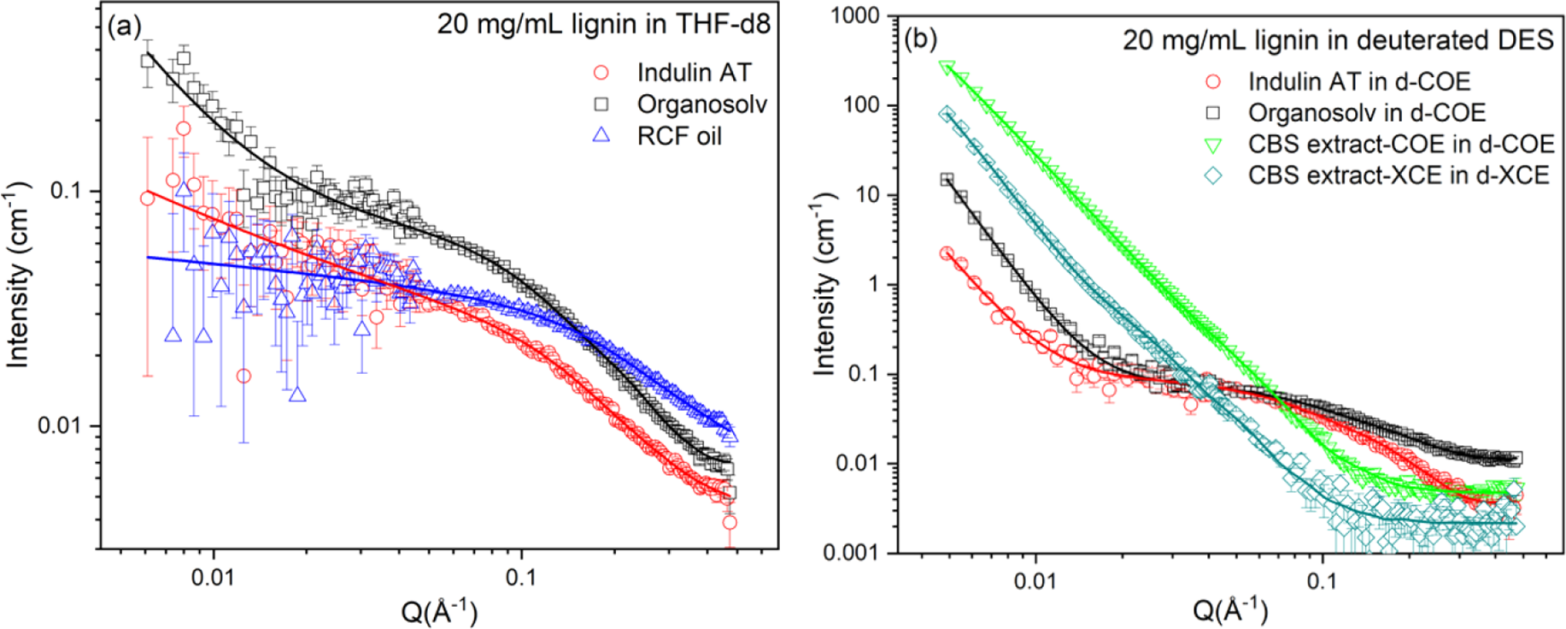
Scattering data from 20 mg/mL solutions of different types of lignin in (a) THF-d8 and (b) deuterated DES. Solid lines are fits to the data sets, as discussed in the text. Error bars corresponding to the measurement uncertainties are included for the experimental data on both graphs but are sometimes smaller than the symbol size.

**Table 1:** Sizes of Lignin Structures in THF-d8 and Deuterated DES (d-COE and d-XCE) from SANS Fitting^a^

20 mg/mL lignin at 25 °C	cylinder radius (Å)	cylinder length (Å)	power exponent	fractal dimension	correlation length (Å)
deuterated THF					
Indulin AT	9.3 ± 0.4	36 ± 2	0.7 ± 0.1		
Organosolv	7.9 ± 0.1	47 ± 1	1.8 ± 0.2		
RCF oil	6.8 ± 0.7	26 ± 2	0.1 ± 0.1		
Deuterated choline chloride/oxalic acid/ethylene glycol DES					
Indulin AT	9.9 ± 0.1	68 ± 2	3.8 ± 0.2		
Organosolv	9.0 ± 0.1	57 ± 2	4.4 ± 0.1		
CBS extract-COE	10 ± 1	68 ± 2		2.8 ± 0.1	501 ± 6
Deuterated xylitol/citric acid/ethylene glycol DES					
CBS extract-XCE	11 ± 5	185 ± 8		3.1 ± 0.1	512 ± 31

a Uncertainties indicate the reproducibility of fitted values, being the standard deviation of these values from at least three independent fits, starting from different initial values.

Although the DES components are functionally effective in dissolving a substantial amount of lignin due to the different intermolecular interactions, this solvent appears to promote aggregation rather than complete molecular dispersion via solvation, resulting in densely packed elongated rod-like morphologies. This behavior aligns with a prior study on kraft lignin in ethylene glycol, which showed partial dissolution and aggregation.^13^ However, the value of power law exponent in DES here is found to be even higher when compared to reported values of lignin in ethylene glycol (2.2). This aggregation behavior can be explained based on the complex solvation environment offered by the DES, composed of ionic components and capable of extensive hydrogen and ionic bonding interactions. These strong interactions are proposed to facilitate chain alignment and stacking, encouraging lignin molecules to form linear, elongated aggregates. Furthermore, the larger molecular sizes, higher viscosity, and polarity of the DES may also be hypothesized to hinder complete solvent penetration, resulting in rigid aggregates, unlike what is observed in more classical moderately polar, small, aprotic solvents like THF.

DES-extracted CBS lignin solutions in DES displayed extremely high scattering intensity, indicating a distinct structural hierarchy and extensive aggregation. Unlike Indulin AT and organosolv in THF and the choline chloride/oxalic acid/ethylene glycol DES, as well as RCF oil in THF, which conform to a power law in the low Q and cylinder model in the high Q, the CBS-extracted lignin in DES (d-COE and d-XCE) was best described by a fractal cylinder model. Such models are well-established in the literature, concerning model-based fitting of SANS data from lignin systems,^6,12,14–16^ and are thought to be indicative of more complex, branched, and hierarchical assemblies rather than simple elongated units. The high correlation length (∼500 Å) for the extracted lignin further suggests large-scale networked aggregation, implying an interconnected fractal network, robust lignin–lignin interactions, and an extended connectivity over long distances.

### 3.2 Effect of D_2_O on Scattering Behavior

The addition of D_2_O into the solvents introduces significant and distinct changes to the molecular organization and solvation of lignin. Previous studies have shown that mixtures of THF and water exhibit a synergistic effect in promoting the dissolution of lignin.^30,31^ Molecular dynamics simulations revealed that these cosolvent systems act as “theta” solvents, by favoring polymer–solvent interactions over self-association. As a result, lignin transitions from a compact, globular structure to an extended, random coil conformation.^32^ This behavior was attributed to the intermediate polarity of THF–water mixtures, which supported the formation of extended well-solvated lignin conformations.^33^ As evident from Figure 2 and Table 2, the effect of D_2_O on real lignin structures in the THF-d8 system is highly dependent on lignin type, arising due to variations in their chemical structure, polarity, and functional group composition.

**Figure 2:**
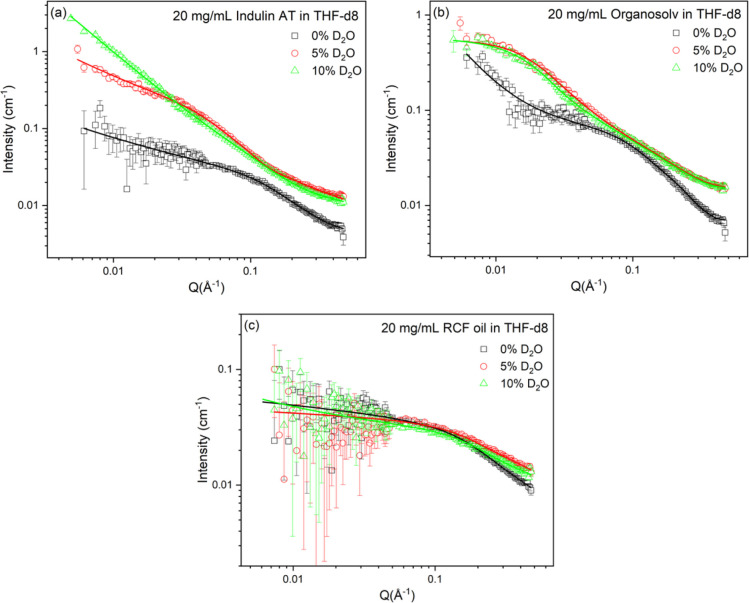
Scattering data from 20 mg/mL solutions of (a) Indulin AT (b) organosolv, and (c) RCF oil in THF-d8 containing different %w/w D_2_O. Error bars corresponding to the measurement uncertainties are included for the experimental data on all graphs but are sometimes smaller than the symbol size.

**Table 2:** Sizes of Lignin Subunits in THF-d8 with Different %w/w D_2_O from SANS Fitting^a^

20 mg/mL lignin at 25 °C	cylinder radius (Å)	cylinder length (Å)	power exponent	Kuhn length (Å)
deuterated THF				
Indulin AT	9.3 ± 0.4	36 ± 2	0.7 ± 0.1	
Indulin AT + 5% D_2_O	23.4 ± 0.3	107 ± 5	1.0 ± 0.1	
Indulin AT + 10% D_2_O	12.5 ± 0.8	49 ± 7	1.5 ± 0.1	
Organosolv	7.9 ± 0.1	47 ± 1	1.8 ± 0.2	
Organosolv +5% D_2_O	7.2 ± 0.1	459 ± 13		88 ± 3
Organosolv +10% D_2_O	7.2 ± 0.1	479 ± 17		129 ± 5
RCF oil	6.8 ± 0.7	26 ± 2	0.1 ± 0.1	
RCF oil +5% D_2_O	3.7 ± 1.3	27 ± 1	0.1 ± 0.1	
RCF oil +10% D_2_O	4.9 ± 1.4	25 ± 2	0.6 ± 0.2	

a Uncertainties indicate the reproducibility of fitted values, being the standard deviation of these values from at least three independent fits, starting from different initial values.

For Indulin AT, the addition of 5 weight % D_2_O into THF leads to about 2.5- and 3-fold increases in the cylinder radius and length, respectively, observed via SANS. This can be attributed to solvent-induced swelling, where D_2_O molecules partially penetrate the lignin aggregates, weakening intermolecular associations and introducing localized hydration shells. This results in chain softening and partial unfolding of the preformed cylinders. This is also evidenced by the increase in the ratio of the power law scale factor to the cylinder model scale factor from 3.2 for Indulin AT in THF to 8.8 with 5% D_2_O, suggesting the increased volume of the aggregates. The greater volume of the scattering objects here leads to an overall increase in total scattered intensity. However, with the addition of 10 weight % D_2_O, this ratio decreases to 3.9, accompanied by a notable reduction in cylinder length, indicating a structural collapse. This can in turn be correlated to the antisolvent precipitation of lignin dissolved in solvents upon the addition of water. The addition of excessive polar D_2_O reduces the solvating power of THF to the extent that lignin becomes less soluble. The polymers then reaggregate into smaller, more compact local forms, which form aggregates, giving low Q scattering, and may even start to precipitate. The increased density and size of these structures means the overall scattered intensity remains similar, although the shape of the scattering curve distinctly evolves as the structures change.

The addition of 5 weight % D_2_O in organosolv lignin solutions dramatically increases the cylinder length by 9.7 times, but the cylinder radius remains unchanged. There is a transition from large aggregates (giving power-law scattering) to flexible solvated rod-like structures that lie within the measurable Q range of the SANS instrument. These data no longer fitted to the power law plus cylinder model but required a flexible cylinder model instead. The lack of a significant change in cylinder radius, despite a huge increase in length, indicates linear fibril formation or alignment of lignin chains, possibly resembling rod-like micelle behavior. Similar one-dimensional elongation of lignin structures was seen by Imel et al.,^16^ where addition of poly­(ethylene oxide) species into DMSO caused elongation of cylindrical structures formed by hardwood lignin subunits. The addition of D_2_O into THF containing organosolv lignin is responsible for structured extension or elongation rather than the collapse seen in Indulin AT. However, with the further addition of D_2_O (10 weight %), there was only a very slight increase in cylinder length, indicating a saturation point where all accessible aggregation sites are already disrupted or hydrated and no further structural extension is energetically favored. Thus, organosolv lignin, which has a chemically less condensed structure than Indulin AT, since it undergoes less repolymerization during initial lignin extraction processes,^34^ is more responsive to D_2_O. This more flexible molecular structure allows significant elongation, forming fibrillar structures rather than just swelling followed by compaction.

Addition of D_2_O to the system containing RCF oil does not show any significant structural change (Figure 2c). This behavior aligns with the relatively low molecular weight and hydrophilic nature of RCF oil, which is well-dispersed and nearly fully solvated in THF. There is no sign of swelling or collapse observed with the addition of up to 10% D_2_O.

Unlike THF, where the addition of D_2_O causes noticeable structural transformations such as elongation, swelling, or even collapse of Indulin AT and organosolv lignin, the scattering pattern (Figure 3a,b) shows that these lignins in choline chloride/oxalic acid/ethylene glycol DES systems respond in a subtle manner. This distinction can be attributed to the fundamental nature of the DES composed of hydrogen bond donors and acceptors that strongly interact with lignin molecules, especially through hydrogen bonding with hydroxyl and carboxyl groups, thereby creating an environment where lignin is already organized into densely packed cylindrical assemblies. The DES environment maintains tight control over lignin organization that is not much disturbed even upon the addition of D_2_O. However, the cylinder dimensions (Table 3) indicate that D_2_O inclusion does promote moderate fibrillar elongation in DES due to swelling.

**Figure 3:**
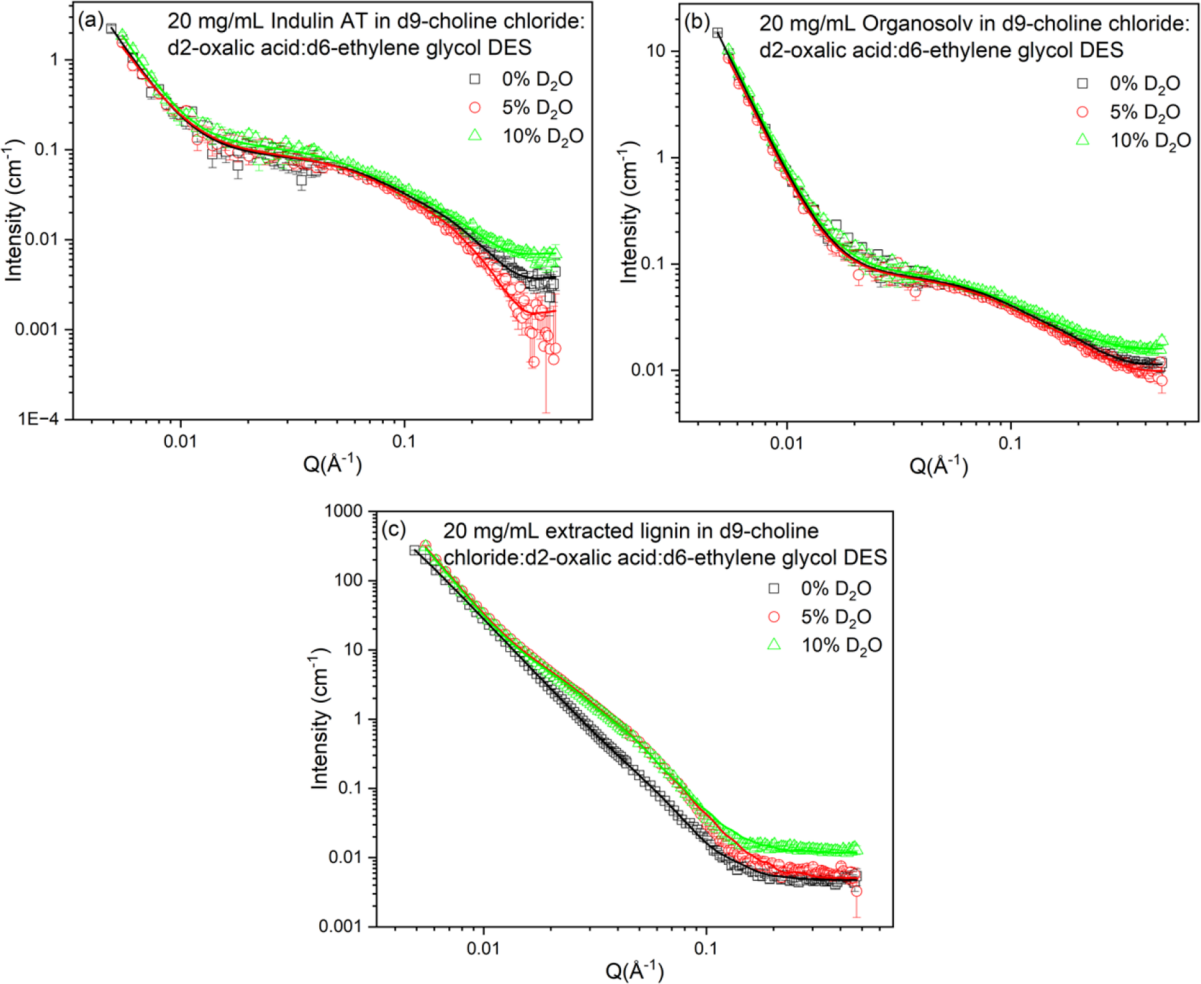
Scattering data from 20 mg/mL solutions of (a) Indulin AT, (b) organosolv, and (c) CBS-extracted lignin in the d9-choline chloride/d2-oxalic acid/d6-ethylene glycol DES containing different w/w D_2_O. Error bars corresponding to the measurement uncertainties are included for the experimental data on all graphs but are sometimes smaller than the symbol size.

**Table 3:** Sizes of Lignin Subunits in the d9-Choline Chloride/d2-Oxalic Acid/d6-Ethylene Glycol DES with Different w/w D_2_O from SANS Fitting^a^

20 mg/mL lignin at 25 °C	cylinder radius (Å)	cylinder length (Å)	power exponent	fractal dimension	correlation length (Å)
deuterated choline chloride/oxalic acid/ethylene glycol DES					
Indulin AT	9.9 ± 0.1	68 ± 2	3.8 ± 0.2		
Indulin AT + 5% D_2_O	10.1 ± 0.1	73 ± 3	3.6 ± 0.2		
Indulin AT + 10% D_2_O	10.6 ± 0.1	83 ± 4	4.0 ± 0.2		
Organosolv	9.0 ± 0.1	57 ± 2	4.4 ± 0.1		
Organosolv +5% D_2_O	8.7 ± 0.1	60 ± 2	4.4 ± 0.1		
Organosolv +10% D_2_O	9.3 ± 0.1	63 ± 2	4.4 ± 0.1		
CBS extract-COE	10.2 ± 1.1	68 ± 2		2.8 ± 0.1	501 ± 6
CBS extract-COE +5% D_2_O	10.4 ± 0.4	216 ± 2		3.0 ± 0.1	661 ± 12
CBS extract-COE +10% D_2_O	10.8 ± 0.4	220 ± 2		3.0 ± 0.1	626 ± 12

a Uncertainties indicate the reproducibility of fitted values, being the standard deviation of these values from at least three independent fits, starting from different initial values.

However, for more highly aggregated DES-extracted CBS lignin, the effect of D_2_O is more pronounced (Figure 3c). This lignin shows a noticeable change in scattering intensity, cylinder dimension (ca. 3 times), and correlation length, indicating that D_2_O enables some degree of unfolding or elongation, possibly by interfering with specific hydrogen bonds or modifying the solvent microenvironment.

### 3.3 Temperature Dependence in DES Systems

The effect of temperature on lignin conformation in the deuterated choline chloride/oxalic acid/ethylene glycol DES was systematically examined, as shown in Figure 4 and Table 4. For Indulin AT, an increase in temperature from 25 to 43 °C leads to fibril elongation and directional alignment. This indicates thermally induced ordering, and the cylinder length reaches 94 Å. However, a further increase to 52 °C results in a sharp structural collapse. This points to a critical thermal stability threshold window beyond which the lignin–DES interactions are no longer sufficient to maintain ordered assembly. In contrast, organosolv lignin exhibits a more moderate elongation of the cylinder length from 57 Å at 25 °C to 71 Å at 52 °C. Unlike Indulin AT, it maintains its structural integrity across this temperature range, implying strong lignin–DES interactions that stabilize its organization. The almost negligible change in the power exponent (∼4.4) across temperatures indicates that the lignin aggregates maintain their rigidity. CBS extract-COE lignin displays a distinctly different thermal response due to its branched and hierarchically assembled structure. Upon heating from 25 to 35 °C, there is a dramatic increase in cylindrical length (from 68 Å to 219 Å), followed by a partial reduction to 151 Å at 52 °C. Simultaneously, the correlation length, indicative of the network’s connectivity, increases significantly from 501 Å to 1095 Å. Despite these changes, the fractal dimension remains nearly constant (∼2.9), implying that while fibrils elongate, the lignin network retains a dense and highly branched architecture. These observations suggest a temperature-driven restructuring process, where aggregates reorganize into longer fibrils without losing the highly branched network.

**Figure 4:**
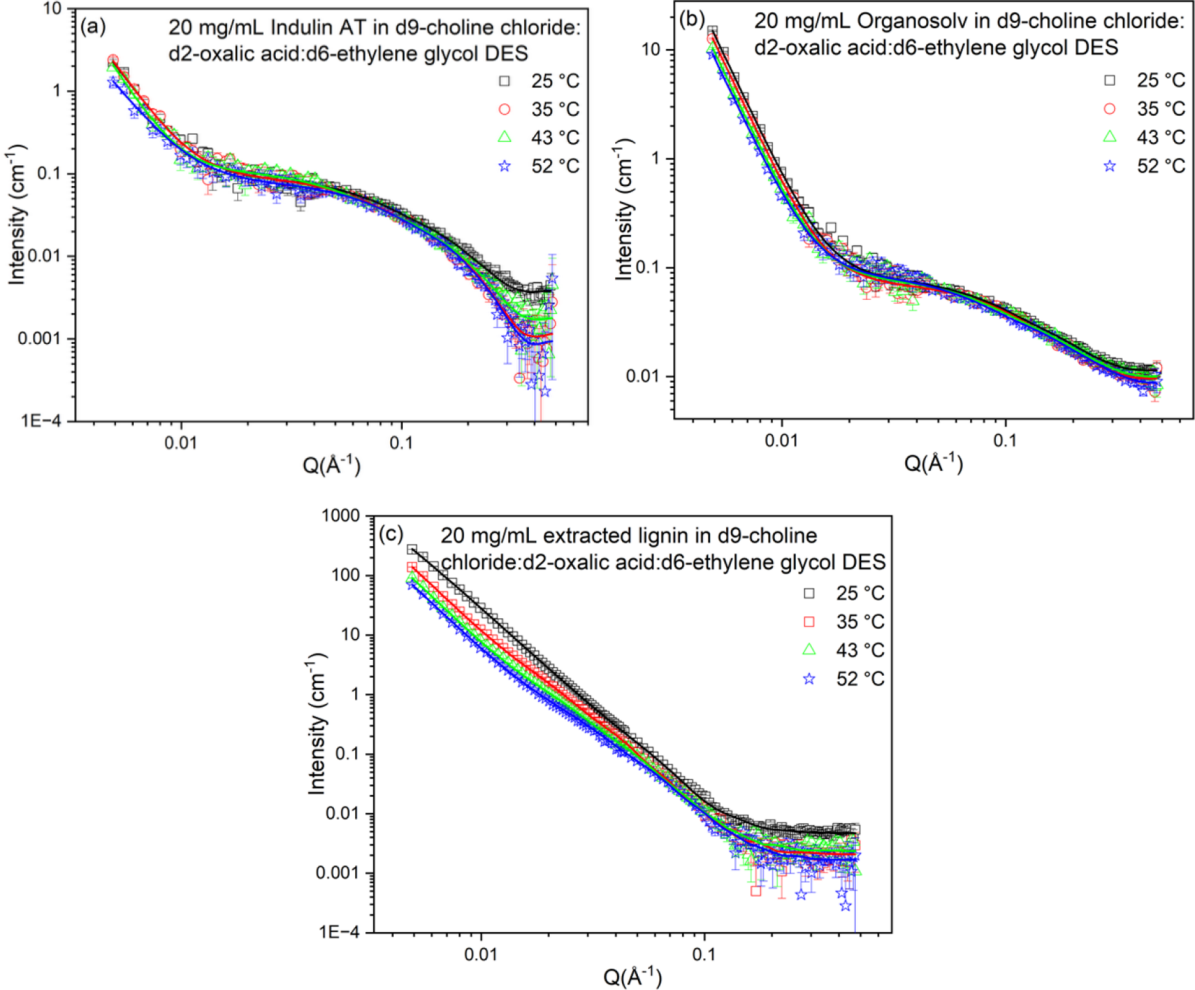
Scattering data from 20 mg/mL solutions of (a) Indulin AT, (b) organosolv, and (c) CBS-extracted lignin in the d9-choline chloride/d2-oxalic acid/d6-ethylene glycol DES at different temperatures. Error bars corresponding to the measurement uncertainties are included for the experimental data on all graphs but are sometimes smaller than the symbol size.

**Table 4:** Sizes of Lignin Subunits in the d9-Choline Chloride/d2-Oxalic Acid/d6-Ethylene Glycol DES at Different Temperatures from SANS Fitting^a^

20 mg/mL lignin	cylinder radius (Å)	cylinder length (Å)	power exponent	fractal dimension	correlation length (Å)
Indulin AT					
25 °C	9.9 ± 0.1	68 ± 2	3.8 ± 0.2		
35 °C	9.7 ± 0.1	76 ± 3	3.8 ± 0.2		
43 °C	9.5 ± 0.1	94 ± 5	4.2 ± 0.2		
52 °C	9.7 ± 0.1	67 ± 3	3.3 ± 0.2		
Organosolv					
25 °C	9.0 ± 0.1	57 ± 2	4.4 ± 0.1		
35 °C	9.1 ± 0.1	52 ± 2	4.4 ± 0.1		
43 °C	8.5 ± 0.1	62 ± 2	4.4 ± 0.1		
52 °C	7.9 ± 0.1	71 ± 3	4.4 ± 0.1		
CBS extract-COE					
25 °C	10 ± 1	68 ± 2		2.8 ± 0.1	501 ± 6
35 °C	7 ± 2	219 ± 4		2.9 ± 0.1	666 ± 32
43 °C	8 ± 2	194 ± 5		2.9 ± 0.1	841 ± 53
52 °C	8 ± 2	151 ± 6		2.9 ± 0.1	1095 ± 106

a Uncertainties indicate the reproducibility of fitted values, being the standard deviation of these values from at least three independent fits, starting from different initial parameter values.

These temperature-induced transformations across lignin types highlight how the interplay between lignin–lignin and lignin–solvent interactions governs structural behavior. Increasing temperature weakens hydrogen bonding and π–π stacking, which can promote elongation or disassembly depending on lignin’s initial conformation and interaction with the DES. Moreover, the addition of 10 weight % D_2_O to the choline chloride/oxalic acid/ethylene glycol DES reduces the temperature-induced changes observed in all three lignin types (Figure S4 and Table S3). This illustrates that the hydrogen-bonding contribution from D_2_O may further stabilize the lignin–DES network and suppresses structural rearrangements.

### 3.4 Structural Correlation of Lignin from SANS and Complementary Techniques

In the study by Papp et al., the molecular weights of the same samples of Indulin AT, organosolv lignin, and RCF oil in different solvents were determined using pfg-diffusion NMR and GPC. The molecular weight obtained for 10 mg/mL lignin samples in THF solvent with pfg-diffusion NMR^11^ was converted into hydrodynamic radius (Rh) using eq 8, assuming spherical geometry

8 $$Rh=\sqrt[{3}]{\frac{3\times M\times \mathrm{\nu ̅}}{4\times \pi \times N}}$$

where Rh is the hydrodynamic (Stokes) radius, *M* is the molecular weight, ν̅ is the partial specific volume, and *N* is Avogadro’s number. This gave the following values: 9.3 ± 0.7, 10.2 ± 0.1, and 7.5 ± 0.2 Å for Indulin AT, organosolv, and RCF oil, respectively.

To investigate whether the SANS data from these samples can be correlated with those measured by pfg-NMR, the Rg for the cylinder dimensions of the lignin subunits was determined using eq 9, for the 10 mg/mL lignin samples measured using SANS (Figure S5a).

9 $${Rg}^{2}=\frac{R^{2}}{2}+\frac{L^{2}}{12}$$

where *R* and *L* represent the cylinder radius and length, respectively, obtained from the SANS fitting parameters. The resulting Rg values: 12.4 ± 0.6 Å (Indulin AT), 14.6 ± 0.6 Å (organosolv), and 7.1 ± 0.8 Å (RCF oil) followed a similar trend to the NMR-derived Rh values, with organosolv showing the highest dimensions and RCF oil the lowest (Figure 5a). Similar Rg values were found for the lignin samples measuring 20 mg/mL lignin in THF (Supporting Information Table S4).

**Figure 5:**
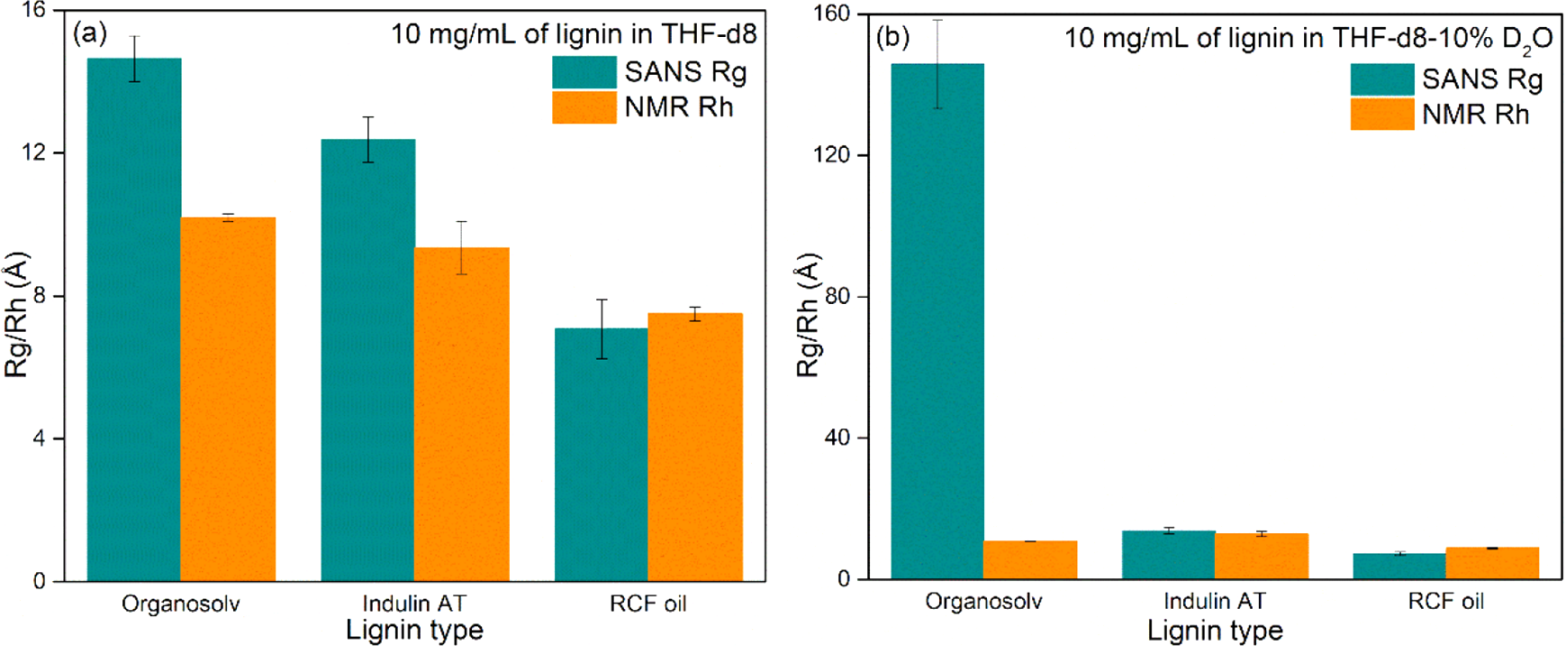
Comparison of the molecular dimensions of three lignin samples (10 mg/mL) measured by pfg-diffusion NMR and SANS in (a) THF-d8 and (b) THF-d8 with 10 weight % D_2_O.

However, a key observation is that for Indulin AT and organosolv lignin, Rg is about 1.3 to 1.4 times larger than Rh, suggesting more elongated and anisotropic structures. The divergence becomes more pronounced with increasing cylinder length. For RCF oil, the close agreement between Rg and Rh indicates a more compact, less elongated structure, likely reflecting its lower molecular weight and diminished intermolecular interactions. This is an expected consequence of hydrogenation during reductive catalytic fractionation, which disrupts cross-linking and π-stacking. DLS measurements (Figure S6) further supported the size trend seen in SANS and NMR but yielded significantly larger Rh values, i.e., 37.7 ± 4.0 Å (organosolv), 24.2 ± 2.1 Å (Indulin AT), and 13.5 ± 1.2 Å (RCF oil). The larger Rh, as observed in DLS and not in pfg-diffusion NMR, reflects the limitations of dynamic light scattering (which is insensitive to objects with radii less than 20 Å^35^) and thus only provides information on the small population of larger aggregates that are present in the system, rather than indicating the presence of an extensive solvation shell in this case.

To gain further insight, pfg-diffusion NMR (Figure 5b) and SANS measurements (Figure S5b) were conducted on 10 mg/mL lignin samples in THF-d8 containing 10 weight % D_2_O. Upon addition of 10% D_2_O to THF, moderate increases in both Rg (from SANS) and Rh (from NMR) were observed for Indulin AT and RCF oil. Indulin AT showed an increase in Rg from 12.4 ± 0.6 to 13.8 ± 0.9 Å and in Rh from 9.3 ± 0.7 to 12.8 ± 0.7 Å, while RCF oil exhibited an increase in Rg from 7.1 ± 0.8 to 7.3 ± 0.5 Å and in Rh from 7.5 ± 0.2 to 8.8 ± 0.2 Å. In contrast, organosolv lignin displayed a striking rise in Rg to ∼146 ± 12 Å, consistent with observations in Section 3.2, whereas its Rh increased only slightly to 10.8 ± 0.1 Å (Figure 5b). This discrepancy suggests that the formation of fibrillar aggregates or flexible extended chain detectable by SANS are not captured by NMR. The loosely packed internal structure of these aggregates may still permit rapid diffusion, resulting in a relatively small Rh observed by NMR. For characterization of lignin solutions, if access to SANS experiments is not possible, pfg-NMR therefore is likely to provide a better estimate of the size of solvated lignin structures than DLS, with caveats for systems that form fibrillar structures.

## 4 Discussion

The solvent-dependent aggregation behavior of lignin observed from SANS profiles and associated fitting parameters can be rationalized in terms of the balance between lignin–solvent interactions and lignin–lignin self-association. Solvents that promote strong, directional intermolecular interactions with lignin, such as hydrogen bonding, ionic interactions, or π–π stacking, do not necessarily favor complete molecular dispersion. Instead, these interactions can stabilize lignin in elongated, densely packed, aggregated morphologies.^13,16^ Such behavior is observed in our study for DES-based lignin systems, which are defined by high polarity, extensive hydrogen-bonding capacity, elevated viscosity, and relatively large molecular components. These features restrict solvent penetration and limit structural relaxation of lignin chains, thereby stabilizing aggregated morphologies. In contrast, smaller, moderately polar, low-viscosity solvents such as THF effectively penetrate lignin structures and disrupt self-association through dipole–π and dispersion interactions, resulting in comparatively smaller and more weakly associated lignin subunits. Similar dispersion behavior has been reported in earlier studies using another small polar aprotic solvent, DMSO-*d*_6_.^12,14^ The influence of cosolvents such as D_2_O can be viewed as a perturbation to the existing solvation environment rather than as a sole factor causing aggregation. By modifying hydrogen-bonding density and effective solvent polarity, cosolvent addition alters the balance between polymer–solvent and polymer–polymer interactions. The resulting structural response depends on the primary solvation network, such that in flexible or weakly constrained environments, cosolvent addition may induce significant conformational rearrangements, as observed for organosolv lignin in THF upon D_2_O addition, whereas in strongly structured solvation environments, the response is generally muted, as seen for Indulin AT and organosolv lignin in DES. Temperature provides an additional means of modulating lignin conformation by weakening hydrogen bonding and aromatic interactions and enhancing chain mobility. Depending on the initial aggregation state and the strength of solvent-mediated interactions, increasing temperature may promote elongation or partial disassembly. In strongly constrained environments such as DES, temperature primarily modulates internal packing within aggregates rather than inducing large-scale conformational transitions.

## 5 Conclusions

This study elucidates the fundamental influence of the solvent environment on the structural dynamics and solvation behavior of lignin, offering molecular-level insights that would improve both analytical characterization and application-oriented lignin processing. Through detailed SANS measurements, we demonstrate that lignin adopts distinct conformations depending on the solvent system: structured, tightly packed cylinders in the diol-based DES and better molecular dispersion in THF. Thus, THF enhances lignin’s suitability for analytical characterization and formulation, whereas the chosen DES holds promise for advanced material applications where hierarchical architecture is desirable. The strong ionic and hydrogen-bonding networks in DES result in highly stable lignin–DES assemblies that show minimal structural changes upon the addition of cosolvents like D_2_O. In contrast, THF exhibits pronounced conformational shifts with D_2_O addition, such as fibrillar elongation, which also varies with lignin type. Temperature-dependent studies further reveal that the DES can support lignin structural ordering, elongation, or collapse within 25–55 °C, depending on lignin type and interaction strength. However, lignin extracted from CBS waste displayed a unique hierarchical structure and a more complex response to D_2_O and temperature variations, likely influenced by its nonwood feedstock origin or the milder extraction conditions employed. Moreover, the Rg values obtained from the SANS data highlight its complementary strength in distinguishing between compact and extended conformations of lignin aggregates, offering crucial insights into lignin’s solution behavior that are not accessible through NMR or DLS alone and thereby improving the accuracy of molecular weight estimation. This work also suggests that pfg NMR gives a better estimate of solvated lignin structures than DLS, given the sizes of lignin species in these solutions. Future work will be directed to understand the influence of diverse DES compositions on lignin structure and self-assembly. Overall, the solvent-dependent solvation and aggregation patterns described here provide fundamental insights that can underpin future strategies for lignin utilization in biorefinery processes and developing high-performance, biobased materials.

## Supplementary Material



## Data Availability

The data associated with the ILL beamtime can be accessed at 10.5291/ILL-DATA.9-11-2222.

## Notes

Neutron scattering length densities for materials used in the study, a schematic showing contributions of power law and cylinder form factor to the fit, a cylinder + power law model fit with the power-law exponent limited to the Porod limit for organosolv lignin, SANS data and fitting parameters from lignin solutions in the COE DES at different temperatures, SAXS data and fitting parameters for 20 mg/mL lignin solutions in COE DES, and SANS and DLS data for 10 mg/mL lignin solutions in THF-d8 (PDF)

## §

Department of Chemical Engineering, Vrije Universiteit Brussel, Brussels 1050, Belgium
