# Supplementary material for: Exploring Lignin Conformation in Organic and Deep Eutectic Solvents Using Small-Angle Neutron Scattering
Source: Langmuir. 2026 Jan 2;42(1):158–69. doi: 10.1021/acs.langmuir.5c03558 (PMC12810389; doi:10.1021/acs.langmuir.5c03558)
Supplement: Supplementary file 1 [file la5c03558_si_001.pdf]

Supplementary Information

Exploring lignin conformation in organic and deep eutectic solvents using small-angle neutron scattering

Subramee Sarkar[a], Maggie Kroon[a], Daniel Papp[a], Nicolas Martin[b], Charlotta Turner[a], Karen J Edler[a*]

[a]*Centre for Analysis and Synthesis, Department of Chemistry, Lund University, Naturvetarvägen 24, Lund, 223 62, Sweden*

[b]*Laboratoire Léon Brillouin, CEA, CNRS, Université Paris-Saclay, CEA Saclay, 91191 Gif-sur-Yvette, France*

Corresponding Author Email: karen.edler@chem.lu.se

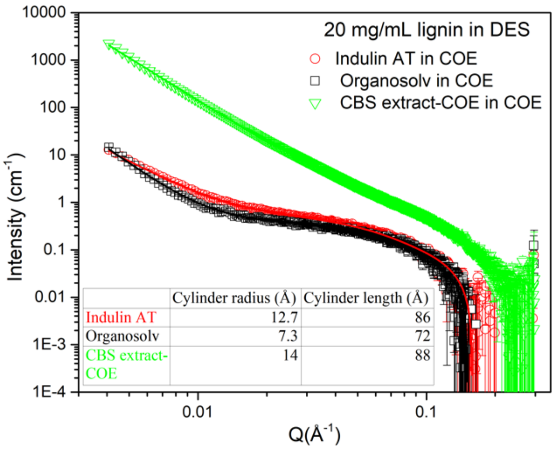

Figure S1: SAXS data on solutions of 20 mg/ml lignin in choline chloride:oxalic acid: ethylene glycol DES at 35 °C, using the same lignin samples as for the SANS experiment, measured in a DES containing only hydrogen containing species. Error bars corresponding to the measurement uncertainties are included for the experimental data on all graphs, but are sometimes smaller than the symbol size.

Table S1: Sizes of lignin structures in choline chloride:oxalic acid:ethylene glycol DES (COE) at 35 °C from SAXS fitting. Uncertainties indicate the reproducibility of fitted values being the standard deviation of these values from at least three independent fits, starting from different initial values.

20 mg/mL lignin at 35 °C	Cylinder radius (Å)	Cylinder length (Å)	Power exponent	Fractal dimension	Correlation length (Å)
Indulin AT	12.7±0.1	86±2	2.6±0.1		
Organosolv	7.3±0.1	72±2	3.4±0.1		
CBS extract-COE	14±1	88±4		2.7±0.1	910±5

The SAXS data from the Indulin AT and organosolv lignin solutions were fitted using power law + cylinder model, while the CBS-extracted lignin was fitted using fractal cylinder model. Fitted cylinder radii and lengths demonstrate that the scattering from these lignins reproduces the SANS results, showing similar numerical values and the same trends. However similar solutions in THF had too low scattering intensity in SAXS measurements to allow fitting to the data, due to the poor electron density contrast.

Table S2: Neutron scattering length densities of materials used in the study.

Material	Chemical formula	Density (g/ml)	SANS SLD ($\times 10^{-6}$ Å^{-2})
THF-d8	C_4D_8O	0.985[a]	6.35[c]
d9-Choline chloride:d2-oxalic acid:d6-ethylene glycol (1:0.2:2)	$C_5H_5D_9NOCl$:$C_2D_2O_4$:$C_2D_6O_2$ (1:0.2:2)	1.13[b]	5.33[c]
d5-Xylitol:d4-citric acid:d6-ethylene glycol (1:1:2)	$C_5H_7D_5O_5$:$C_6H_4D_4O_7$:$C_2D_6O_2$ (1:2:2)	1.37[b]	4.58[c]
Deuterium oxide	D_2O	1.11[a]	6.39[c]
Lignin	$[C_9H_{10}O_3(OCH3)_{0.9–1.7}]_m$	1.5[d]	1.8[d]

[a]Data obtained from the Material Safety Data Sheet available on the Sigma-Aldrich website.
[b]Density measured using an Anton Paar DMA 4500 M at 25 °C.
[c]SLD calculated using the SASView SLD calculator.
[d]SLD and mass density of lignin obtained from references [1, 2]

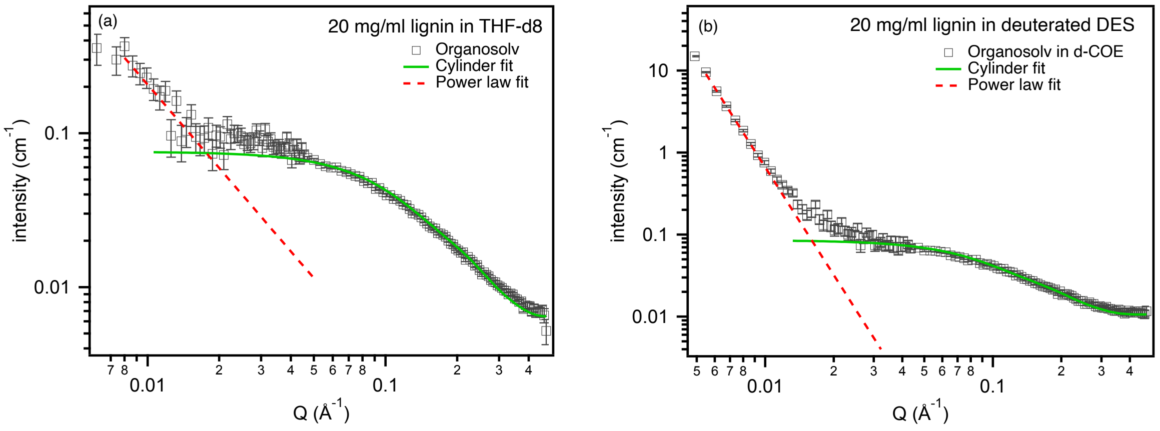

Figure S2: SANS profiles of organosolv lignin in (a) deuterated THF and (b) d9-choline chloride:d2-oxalic acid:d6-ethylene glycol, where solid lines represent contribution to the fits from the cylindrical form factor and dashed lines correspond to the power law model. Error bars corresponding to the measurement uncertainties are included for the experimental data on both graphs, but are sometimes smaller than the symbol size.

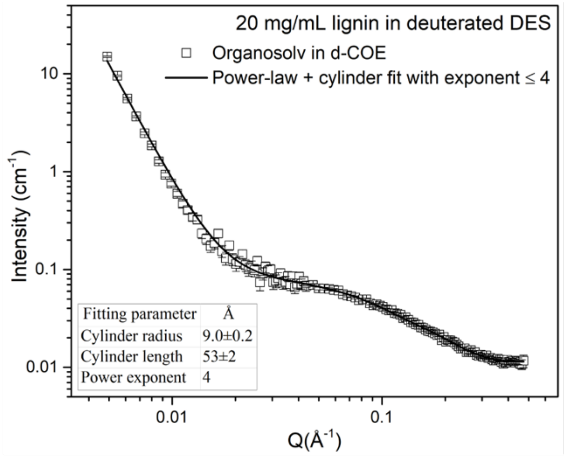

Figure S3: SANS profile of organosolv lignin in deuterated d9-choline chloride:d2-oxalic acid:d6-ethylene glycol fitted using cylinder + power law model, with the power-law exponent constrained to a maximum value of 4 (Porod limit). Error bars corresponding to the measurement uncertainties are included for the experimental data on both graphs, but are sometimes smaller than the symbol size.

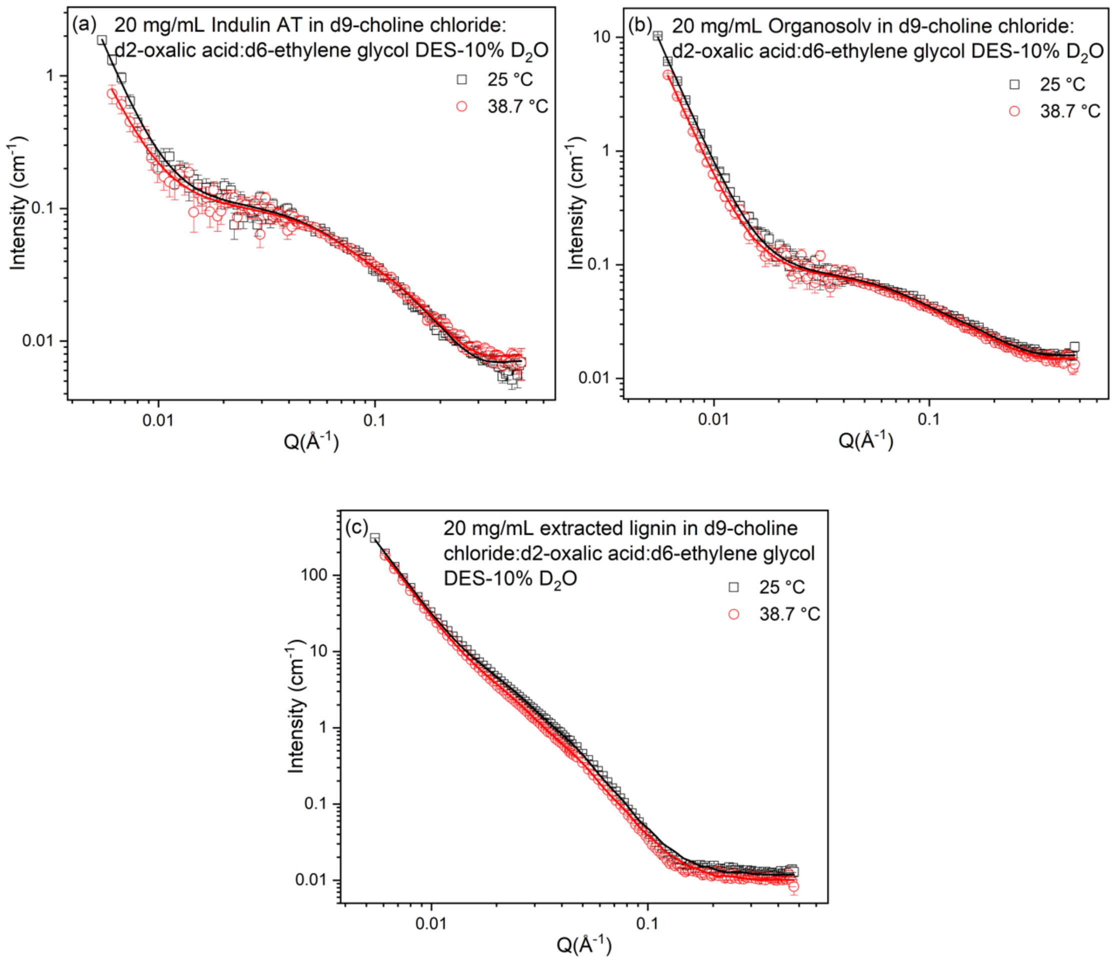

Figure S4: Scattering data from 20 mg/mL solutions of (a) Indulin AT (b) organosolv and (c) CBS-extracted lignin in d9-choline chloride:d2-oxalic acid:d6-ethylene glycol DES with 10 weight% D_2O at different temperatures. Error bars corresponding to the measurement uncertainties are included for the experimental data on all graphs, but are sometimes smaller than the symbol size.

Table S3: Sizes of lignin subunits in d9-choline chloride:d2-oxalic acid:d6-ethylene glycol DES having 10% w/w D_2O at different temperatures from SANS fitting. Uncertainties indicate the reproducibility of fitted values being the standard deviation of these values from at least three independent fits, starting from different initial values.

20 mg/mL lignin	Cylinder radius (Å)	Cylinder length (Å)	Power exponent	Fractal dimension	Correlation length (Å)
		Indulin AT + 10% D_2O			
25 °C	10.6±0.1	83±4	4.0±0.2		
38.7 °C	10.7±0.1	79±4	3.6±0.4		
		Organosolv + 10% D_2O			
25 °C	9.3±0.1	63±2	4.4±0.1		
38.7 °C	9.3±0.1	64±3	4.3±0.1		
		CBS extract-COE + 10% D_2O			
25 °C	10.8±0.4	220±2		3.0±0.1	626±12
38.7 °C	9.5±0.6	213±2		3.0±0.1	548±13

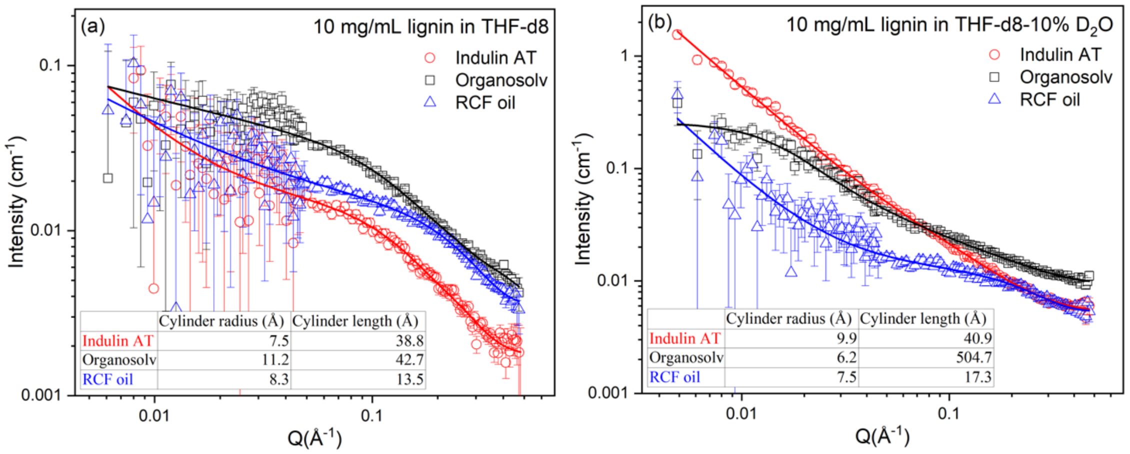

Figure S5: Scattering data from 10 mg/mL solutions of different types of lignin in (a) THF-d8 and (b) THF-d8 with 10 weight% D_2O. Error bars corresponding to the measurement uncertainties are included for the experimental data on all graphs, but are sometimes smaller than the symbol size.

Table S4: Comparison of the Rg values obtained from SANS data at 10 mg/mL and 20 mg/mL samples.

Lignin	10 mg/mL Rg (Å)	20 mg/mL Rg (Å)
Indulin AT	12.4	12.3
Organosolv	14.6	14.7
RCF oil	7.1	8.8

For Indulin AT and organosolv lignin, the Rg values remain essentially unchanged. However, RCF oil shows a slightly higher Rg at 20 mg/mL, likely due to weak concentration-induced aggregation at higher lignin loading.

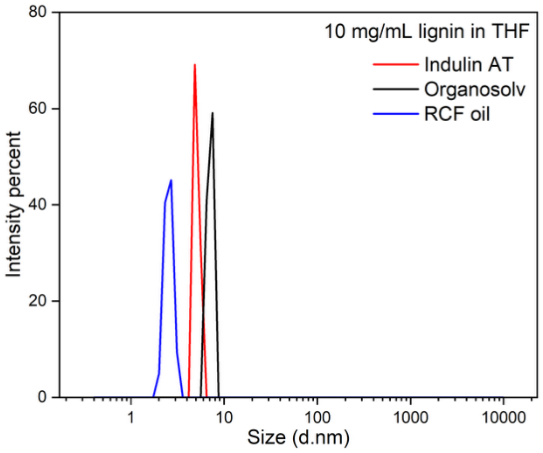

Figure S6: DLS data from 10 mg/mL solutions of Indulin AT, RCF oil and organosolv lignin in THF.

References

(1) Cheng, G.; Zhang, X.; Simmons, B.; Singh, S. Theory, practice and prospects of X-ray and neutron scattering for lignocellulosic biomass characterization: towards understanding biomass pretreatment. *Energy Environ. Sci.* **2015**, *8* (2), 436–455, DOI: 10.1039/C4EE03147D.

(2) Zhao, W.; Xiao, L.-P.; Song, G.; Sun, R.-C.; He, L.; Singh, S.; Simmons, B. A.; Cheng, G. From lignin subunits to aggregates: insights into lignin solubilization. *Green Chem.* **2017**, *19* (14), 3272–3281, DOI: 10.1039/C7GC00944E.
